# Physicochemical and Sensory Parameters of “Petipan” Enriched with Heme Iron and Andean Grain Flours

**DOI:** 10.3390/molecules28073073

**Published:** 2023-03-30

**Authors:** Nicodemo C. Jamanca-Gonzales, Robert W. Ocrospoma-Dueñas, Norma B. Quintana-Salazar, Jose N. Jimenez-Bustamante, Eduardo E. Herrera Huaman, Reynaldo J. Silva-Paz

**Affiliations:** Departamento de Ingeniería—Escuela de Ingeniería en Industrias Alimentarias, Universidad Nacional de Barranca, Av. Toribio de Luzuriaga N° 376 Mz J-Urb. La Florida, Barranca 15169, Peru

**Keywords:** enriched, petipan, texture, iron, color

## Abstract

Enrichment is the addition of nutrients to a food that does not contain them naturally, which is conducted in a mandatory manner and in order to solve a nutritional deficiency in the population. Enriched petipan are products that contain heme iron. The objective of this research was to evaluate the physical, chemical, mechanical and sensory characteristics of petipan produced with Andean grain flours and heme iron concentrate. A completely randomized design (CRD) with five experimental treatments was used with different levels of heme iron. The results show the direct influence of the heme concentration level on the chromatic, mechanical and textural characteristics of petipan. As the heme concentrate increases, its mechanical properties are considerably affected, with there being a correlation between the color intensity and a considerable reduction in its porosity. Samples without heme iron (T0) and 1% heme iron concentrate (T1) present the best mechanical and sensory characteristics; however, the incorporation of heme concentrate directly influences its nutritional, textural, and mainly chromatic components.

## 1. Introduction

One way to help overcome child malnutrition and micronutrient deficiencies in women is by using different strategies, such as dietary diversification, supplementation, biofortification and food fortification, including substances that are rich in iron, folate, zinc, protein, β-carotene, calcium, potassium, and fiber as a possible natural enrichment or fortifier [1]. Not all foods provide heme iron, and its absorption capacity is highly variable, with availability in the diet being a very influential factor; likewise, food fortification and enrichment are considered the most profitable and economically attractive than iron supplementation [2]. Food fortification or enriched hemoglobin is a way to take advantage of heme iron [3], requiring an enhancer such as ascorbic acid that can increase its absorption [4].

Bread is a food for daily consumption, defined as a perishable product resulting from cooking a dough obtained by mixing wheat flour, edible salt and drinking water and fermented by species typical of bread fermentation, such as *Saccharomyces cerevisiae* [5]. Within the range of bakery products, we find “petipan” to be characterized by being a small bread that is widely consumed in social gatherings as a snack [6,7]. Furthermore, it is ideal to be used with substitute flours such as quinoa, kiwicha or other cereals such as rice; these are likely to be incorporated from inputs that increase their nutritional value, mainly with inorganic micronutrients such as iron. However, this may mean that there is little acceptability of the product due to its sensory changes, mainly in color, which are necessary for the growth and development of children [8]. Proposals for the enrichment of bakery products have been developed to promote the growth of children, such as sliced bread made from red beans *(Phaseolus vulgaris)*, soybeans *(Glycine max)* and y corn *(Zea mays)*, which are classified as functional due to their useful nutritional components such as amino acids, proteins, vitamins and minerals that are effective for health, physical growth and brain development [9]. Substitution can cause physical, chemical, textural and sensory changes, as well as the incorporation of tarwi flour *(Lupinus mutabilis)* (0–20% *p*/*p*), yeast extract (0–2% *p*/*p*), guar gum (0–0.20% *p*/*p*), increased protein content, water absorption, and dough pH. However, high levels of tarwi flour can cause a reduction in specific volume; with a substitution of 20% (*w*/*w*), a reduction of 8.33% was observed. Moreover, the addition of guar gum at 0.15% (*w*/*w*) increased the specific volume [10]. Additionally, the partial substitution with quinoa flour and tarwi generated similar thermomechanical and textural properties in the dough and in sliced bread.

The increase in tarwi flour shows a negative effect on bread, mainly its specific volume, crumb porosity, and textural properties. A negative technological impact of the high percentages of substitution of wheat flour by a mixture of tarwi and quinoa flours was found, but this contrasted with a positive effect on nutritional quality, particularly evidenced by a high protein and dietary fiber content [11]. In bread made with flour combined with the incorporation of chia, quinoa and amaranth, the specific volume decreased slightly; however, they were characterized as gluten-free products and a large amount of fiber [12]. The incorporation of kiwicha in bread (from 10 to 40%) increases moisture, protein, fat, fiber and anti-nutritional content, and this increase influences the sensory aspect [13]. Therefore, the use of amaranth flour by up to 10% is an alternative to improve its nutritional value and improve its physical characteristics and proximal composition, especially proteins, lipids, minerals, and high digestibility [14]. Heme iron concentrate has a greater absorption capacity than non-heme iron and has been used in the production of milk fortified with heme iron from bovine hemoglobin hydrolysates [15].

Both substitute flours and enrich substances that have an impact on the physical and sensory characteristics of the final product (bread), specifically on liking, influencing its appearance, flavor, and general taste, which in the case of bread is due to its darkening and residual taste. At present, fast sensory evaluation methodologies are being applied and worked with consumers such as CATA (Check All That Apply), JAR (Just All Right), RATA (Rate All That Apply), Sorting, Napping, Pivot Profile, etc. [16]. Iwamura et al. [17] applied JAR to evaluate the sensory profile of gluten-free bread prepared with teff *(Eragrostis tef)*, sorghum *(Sorghum bicolor)* and yacon *(Smallanthus sonchifolius)* flours. Sweet and salty flavored attributes were considered above and below the ideal, respectively, although they are considered attributes to take into account, as well as softness for bread. In addition, CATA and JAR tests can be used to compare samples with subtle differences in their attributes [18]. The mixture of these flours contributes to obtaining gluten-free bread with better sensory characteristics than when these flours are used alone. On the other hand, the Pivot Profile test has been used in the sensory evaluation of artisan pasta as an alternative to describing food with consumers [19].

As described above on the use of substitute flours and/or bread enrichment, the objective of this study was to elaborate and evaluate the physicochemical characteristics of petipan when produced with five levels of heme iron concentrate obtained from bovine blood, incorporating quinoa *(Chenopodium quinoa)* and kiwicha *(Amaranthus caudatus)* flour, and evaluating the physicochemical parameters, composition proximal, mechanical and chromatic properties.

## 2. Results and Discussion

### 2.1. Analysis of the Centesimal Composition

Table 1 shows significant differences (*p* ≤ 0.05) in all the percentage compositions of the petipan, with the exception of fat and energy value (*p* ≥ 0.05). Regarding the carbohydrate content, T0, T1, and T2 presented the highest carbohydrate contents, which was statistically similar between them, although T3 and T4 registered lower values. Regarding the ash content, no marked trend was observed with the increase in iron samples T1, T0 and T4 were similar to each other (*p* ≤ 0.05), and these last two samples did not present significant differences to T2 and T3. Humidity was higher for T0, T3 and T4 (*p* ≤ 0.05) and lower for T1 and T2. The variations in humidity were attributed to the incorporation of heme iron, which allowed the characteristics of the petipan under storage, packaging, temperature and relative humidity conditions to be maintained. These results coincide with those reported by Calderon et al. [10] and Zula et al. [13]. Therefore, the higher the concentration of heme iron, the higher the humidity (Table 2). In addition, it is observed that the lower the humidity, the more the total solids content increased, including mainly protein. The protein content increased with the presence of the iron concentrate, producing a lower content with the absence (T0) and an increase in a higher content (samples T1 to T4), demonstrating that the influence was directly proportional to the protein content since heme iron concentrate (total carbohydrates 11.33 ± 0.72%, ash 3.31 ± 0.06%, fat 33.33 ± 0.02%, moisture 5.25 ± 0.07%, protein 77.29 ± 0.01%, total energy 382.41 ± 0.08 Kcal/100 g and iron content 2447.65 ± 33.73 mg/kg), were obtained from cattle blood, which is an important source of hemoglobin [20].

### 2.2. Analysis of Iron Content and Physical Parameters

In Table 2, it was observed that the iron content, pH, acidity, and porosity presented significant differences (*p* ≤ 0.05) between the treatments. A higher iron content occurred with an increase in the percentage of heme iron concentrate (T0 < T1 < T2 < T3 < T4). The decrease in pH was observed as the percentage of the heme iron concentrate increased; although, in acidity, it showed an inverse behavior produced by acidification during the enzymatic hydrolysis treatment prior to obtaining the heme iron concentrate [21]. Porosity showed a behavior similar to that of the pH, which decreased as the heme iron concentrate increased, reducing the swelling and sponginess of gluten: a behavior described in the bread obtained with the addition of freeze-dried kale *(Brassica oleracea L. var. sabellica)* in powder [22] and the addition of mesquite flour *(Prosopis juliflora)* with a lower volume and porosity [23]. The apparent density and specific volume did not show significant differences (*p* ≥ 0.05) between the treatments; this behavior was reported by Jamanca et al. [24] in pre-fermented panettone with quinoa and kiwicha.

**Figure 1:**
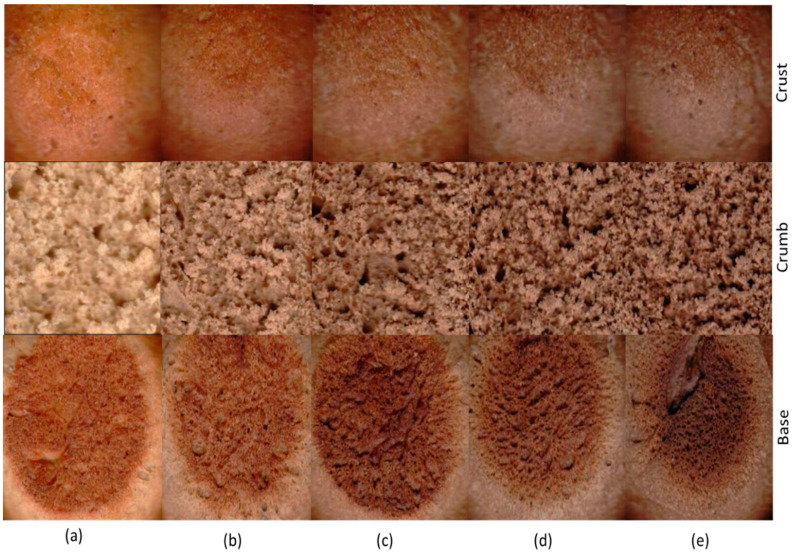
Images of petipan structure: rind, crumb and base (0.025 × 0.025 m image area). (**a**) T0, (**b**) T1, (**c**) T2, (**d**) T3 and (**e**) T4.

Baking products are structured and have porous crumbs and regular cells. However, the incorporation of the heme iron concentrate influences the formation of cells or alveoli (Figure 1), also known as holes in the crumb, which are caused by the air that is trapped between the gluten networks. Although there are no standardized parameters that are applied to bakery products (size, volume and thickness), since these parameters would allow bakery products to be characterized, they should still be consistent and undergo reformulations that generate volumetric expansions during baking [25,26]. Petipan samples presented a highly variable porous crumb structure. The bark and base of the petipan showed a marked difference in terms of color tonality (brown or white surfaces and bases). In baked goods, it is difficult to obtain homogeneous surfaces and crumbs (porosity) due to the heterogeneous distribution of heat during baking and the Maillard reaction [27].

### 2.3. Colorimetric Parameters of Enriched Petipan

Colorimetric data are shown in Table 3; significant differences (*p* ≤ 0.05) were observed in all the parameters on the scale of the international color system, including both L*, a*, b*, C* and h* in the crust, crumb and base of the petipan. At the level of the crust, the luminosity “L” (between black and white) was higher for T0, corresponding to the sample without haem iron concentrate, which is very close to whole wheat bread prepared from white durum wheat and red durum wheat [28,29] in the parameters L* and b*. However, the base registers a lower luminosity (darker) than the crust from the addition of 3% concentrated haem iron (sample T3), and the luminosity of the crust approaches the crumb. The higher the percentage of the concentrate, the lower the luminosity (the sample darkens more intensely), which is intensified by the Maillard reaction that occurs between sugars and proteins during baking, forming melanoidins, which are the final compounds of the said reaction, giving it the color that is characteristic dark brown [27]. The parameter a* presents significant differences (*p* ≤ 0.05) among all the treatments, whose positive values present a decreasing tendency in the dark red tone when the level of heme iron concentrate increases, while the parameter b* with a decreasing tendency and yellow tone when the level of iron concentrate increases. The same trend observed in the crust was maintained in the crumb and the base.

### 2.4. Instrumental Texture Analysis

Mechanical parameters are important in bakery products. Table 4 presents the results of the instrumental texture profile, finding significant differences (*p* ≤ 0.05) in hardness, fracturability, cohesiveness, elasticity and chewiness, while in adhesiveness, there were no significant differences (*p* ≥ 0.05). Sample T4 presented lower values in resilience, cohesiveness and elasticity compared to the other samples. However, this same sample presented higher values of hardness, fracturability and chewiness. The hardness or softness of the crumb is a property that receives more attention in the evaluation of bakery products due to its close association with the human perception of freshness [30]. Lower moisture is related to stale and dry bakery products that require more salivation and chewing, although chewiness is directly related to the presence of fat and sugar [31]. Some ingredients, such as flour, sugars, fats, emulsifiers, enzymes, gluten and flour improvers, together with the humidity of the dough and storage (processing time and packaging of the product), affect the quality of the final product [32]. These values were in the ranges reported by Guiné [33] and in molded bread in relation to the texture profile.

### 2.5. Sensory Analysis

#### 2.5.1. Pivot Profile

**Figure 2:**
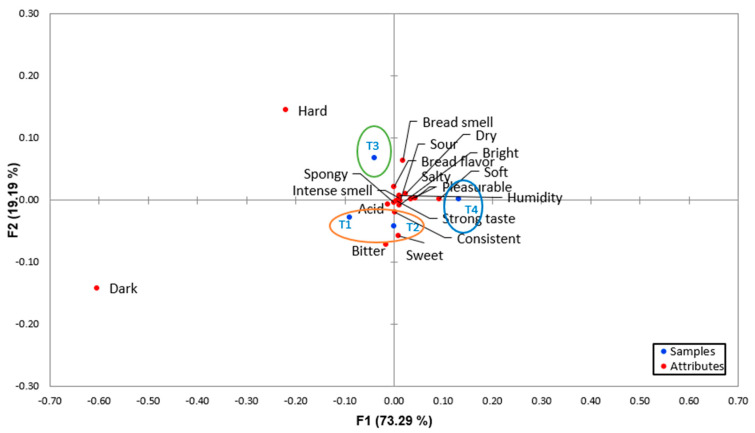
Correspondence analysis of the contingency table obtained from the pivot profile.

The results of the descriptors obtained with the pivot profile for each of the samples were analyzed, grouped by categories, and summarized in a contingency table (Table 5). In addition, the sensory map was developed through correspondence analysis (Figure 2). Of the total thirty-four descriptors, sixteen did not exceed a 20% frequency of use. Eighteen descriptors represented 53% of the terms used and were classified into four categories, as follows: eight terms for flavor (acid, pleasurable, sour, bitter, sweet, bread flavor, strong taste and salty), two terms for the visual appearance of the bread (dark, bright), five terms for the texture (consistent, hard, spongy, humidity, dry and soft) and two terms for the smell (bread flavor and intense smell).

The first two dimensions of the correspondence analysis explained 92.48% of the variability of the total data. Three groups were observed: in the first group, samples T1 and T2 were characterized by being acidic, consistent, sweet, bitter, bright and spongy, which contrasted with the physicochemical and textural characteristics. In the second group, sample T3 was described as having a bread flavor and being hard; in group three, sample T4 was characterized by humidity and a soft texture. The dark attribute was valued as a characteristic inherent to all the samples, unlike T0, which presented a bright color compared to all the samples.

To specifically identify associations between descriptors and samples, the chi-square cell-by-cell test was performed [34]. From the contingency table (Table 5), making an evaluation by pairs or groups [35] meant that a significant influence (*p* ≤ 0.05) was found for the dark and hard descriptors. In terms of visual appearance, the T1 sample was considered less dark, and the T4 sample was darker. The T3 sample was less hard, and the T4 sample was harder. There were no significant differences (*p* ≥ 0.05) between the samples regarding the descriptors of acidity, pleasure, sour, bitter, sweet, bread flavor, strong taste, salty, bright, consistent, spongy, humidity, dry, soft, bread and intense smell.

#### 2.5.2. Just about Right (JAR)

**Figure 3:**
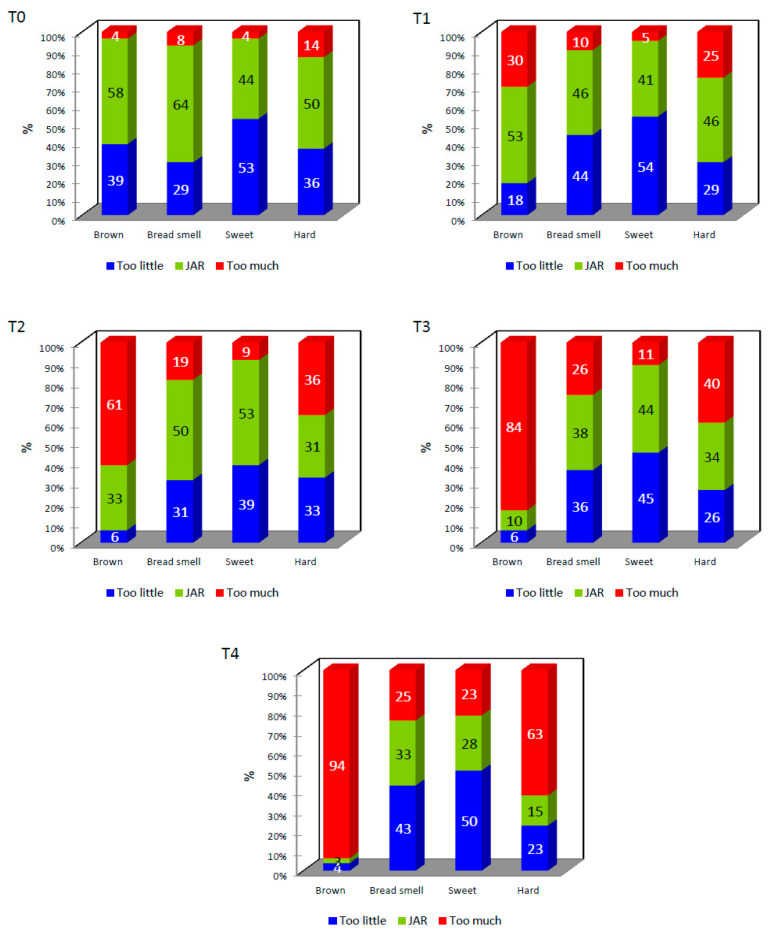
Representation of the % of consumers that selected the different levels in the JAR scale for each sample.

The Just About Right (JAR) test, known as the ideal point scale [18], was used to identify whether the four basic attributes: brown (color), the smell of bread (smell), sweet (taste) and hardness (texture) determine if the product is in the acceptable area or, on the contrary, if they needed to increase or reduce its intensity (optimization). Figure 3 shows the graphs obtained by the JAR method.

**Figure 4:**
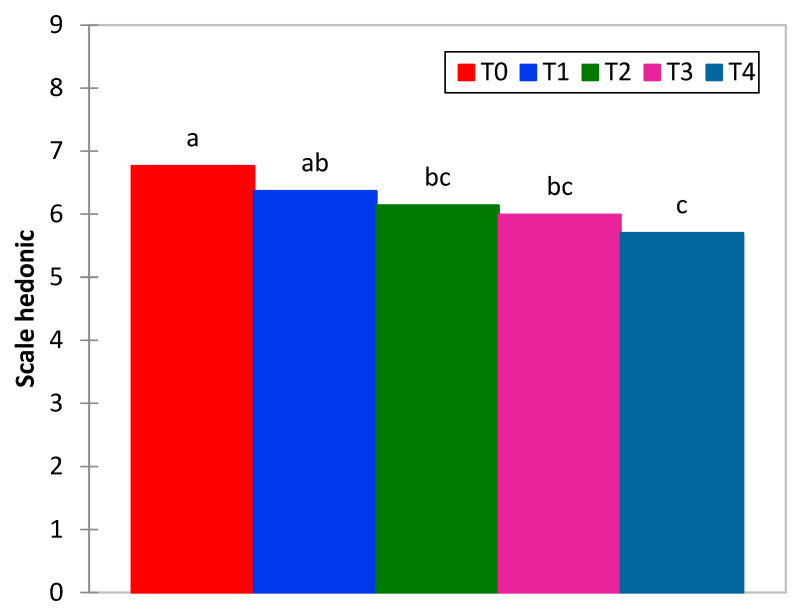
Overall liking of petipan using a 9-point hedonic scale.

Figure 4 shows the results of the acceptability test. Samples T0 and T1 presented greater acceptability (these samples are similar to each other, *p* ≥ 0.05) and were statistically different from samples T2, T3 and T4 (there were no significant differences between these samples, *p* ≥ 0.05), which showed less liking. The sample without addition and a low percentage (1%) of heme iron concentrate allowed for the adequate acceptability of consumers. However, the increase in concentrate led to a decrease in the acceptability of the product, which could ultimately lead to the rejection of enriched petipan.

## 3. Materials and Methods

### 3.1. Raw Material of Enriched Petipan

For the preparation of enriched petipan, the ingredients were incorporated according to a base formulation consisting of 43.47% wheat flour, 2.71% quinoa flour, 8.15% kiwicha flour, 0.55% improver, 1.63% powdered milk, 2.45% yeast, 14.67% water, 0.82% salt, 8.15% sugar, 7.09% margarine, 1.63% vanilla essence, 1.08% honey, and 7.60% egg. For this, a completely randomized design (DCA) was used with five levels of heme iron (0%, T0; 1%, T1; 2%, T2; 3%, T3; and 4%, T4).

The raw material and inputs were purchased in the food market of the city of Barranca. The heme iron concentrate provided by Camal Conchucos S.A.—Lima—Peru consisted of a concentrate from bovine hemoglobin.

### 3.2. Elaboration Process of Enriched Petipan

An elaboration of petipan was carried out in the baking laboratory of the National University of Barranca—Peru; it consisted of mixing the inputs in two stages: first, the powdery dry inputs (wheat, quinoa, kiwicha, improver and powdered milk) were mixed, and the second stage consisted adding the wet inputs to the previous mix and granulated solids, in the following order: water, white sugar, honey, yeast, egg, vanilla essence, margarine, and salt. Finally, the entire mixing and kneading process was carried out at a speed of 18 rpm for 30 min in an industrial kneader–kneader (25 kg Nova brand, model K-25, Peru) until it was consistent, smooth and elastic. The dough rested for 10 min, then we proceeded, and the prepared dough of 1000 g was divided into 55 units of 18 ± 1 g into portions. Subsequently, it was rounded and placed on previously greased metal trays. The products were taken to a fermentation chamber (Nova brand, model Max 1000, Peru) for 1.5 h at 28 °C with a relative humidity of 80–85% and were then baked at 160 °C for 6 min in a gas rotary oven (Nova brand, model Max 1000, Peru). The product was cooled at room temperature (approximately 18–20 °C) for 1 h; then, it was bagged in medium-density polypropylene containers, sealed with a sealing machine, and labeled and stored in cardboard boxes in a cool environment, according to the indications reported by Mesas and Alegre [5].

### 3.3. Physicochemical Analysis

The physicochemical analyses that were carried out were: Ash, using a muffle (Thermo Concept, model KL15/11) at 500 °C until achieving a constant weight (approx. 3 h) [36], fat in the Soxhlet equipment using a 5 g sample [37], humidity with an oven (Binder, model FD53) at 105 °C for 3 h and dry matter by weight difference [38], protein using the Kjeldahl method with its three stages: digestion with concentrated sulfuric acid and catalytic mixing. This was followed by hot distillation with sodium hydroxide and subsequent titration with sulfuric acid; for the final calculation, the factor 6.25 was applied [39], and the total carbohydrates were calculated by difference [40] and iron content using the atomic absorption spectrophotometric method [41]. The total energy was calculated using the conversion factors recommended by the FAO [40]. The physical-chemical analyses applied to the petipans samples were carried out in triplicate from 30 units.

### 3.4. Analysis of Product Porosity

Porosity was determined by image analysis. Images were obtained with the Laser Jet Pro Scanner (Model 400 MFP, Serie M425 DN), using a black background cover (30 cm × 25 cm), natural lighting and a 300× zoom. The brightness, contrast and threshold of the images were adjusted and converted to 8-bit binary images for analysis using the ImageJ^®^ Software particle analysis tool (https://imagej.nih.gov/ij/ (accessed on 12 December 2022)). The porosity percentage was calculated as the ratio between the total pore area and the total area of the image [42,43].

### 3.5. Color Parameters

The color parameters of the crust, crumb and base were quantified with the 3NH Colorimeter (10° observation angle, Illuminant D65 at 420 nm). The color was recorded using the CIE-Lab system, which used a uniform color space, where L* is lightness, a* indicates the hue on a green (–) to red (+) axis, and b* is the hue on a blue (–) to yellow (+) axis [44]. Additionally, C* and h* (C* is the chroma, and h* denotes the hue or angle of a polar measurement) were determined. The chroma value was calculated C* = (a*2 + b*2)^0.5^ and hue angle h* = arctangent (b*/a*) [45,46]. The color was determined to quadruplicate at room temperature.

### 3.6. Instrumental Texture Profile

Texture profile analysis (TPA) parameters were determined using a Brookfield Texturometer (model CT34500, serial 8555940, Middleboro, MA, USA) and Brookfield Engineering Labs Inc.’s TexturePro CT V1.4 Build 17 software. The test conditions were: TA25/100 compression plate probe, double compression, speed 0.5 mm/s, deformation 40%, load cell 4.5 kg, evaluating hardness, cohesiveness, elasticity, adhesiveness, resilience, fracturability, and chewiness [47]. The samples were measured in their entirety (42–45 mm in diameter, with a height of 15–20 mm in thickness), taking the measurements as quickly as possible to avoid moisture loss. Texture parameters were measured in triplicate at room temperature and at relative humidity.

### 3.7. Sensory Analysis

#### 3.7.1. Consumers

The sensory study was developed with 80 consumers between 19 and 45 years old (46.25 and 53.75% male and female, respectively). All the participants regularly consumed baked goods. The participants were students, professors and administrators of the University National of Barranca. This work was conducted by the Food Analysis Laboratory at relative humidity and room temperature. The samples were presented monadically per sample unit. The consumers ate loaves on a regular basis. The participants performed descriptive and affective tests of the enriched petipan in two sessions (5 samples per session). For their participation, their informed consent was requested, which was approved by the ethics committee with code No. 005–2022-UNAB/CEPI.

#### 3.7.2. Descriptive Test—Pivot Profile

For the Pivot Profile, a pair of randomly selected samples was delivered (one identified as a reference and the other code with a three-digit numerical code). For each pair of samples, they were first asked to taste both samples and then record (with their own vocabulary) the attributes that could describe the sample (appearance, color, smell, taste, texture) that they perceived more or less intensely compared to the reference sample. It was indicated to drink water after the evaluation of each sample to rinse the palate [48,49,50].

#### 3.7.3. Affective Test—Just about Right (JAR)

Samples were cut in half (9 ± 1 g) and presented in 0.1 m diameter polypropylene (P15) dishes. The samples were coded with three random numbers. The evaluation was carried out at room temperature in individual cabins. Each panelist was given the evaluation form where the attributes were randomized, and they were asked to rate the sensory characteristics (brown color, smell of bread, sweetness, and hardness) of the petipan on a 5-point scale using the JAR (from 1 = much less to 5 = much more) and overall liking with 9-point hedonic scale (from 1 = extremely dislike to 9 = extremely like with an intermediate value of 5 = neither like nor dislike) [51,52].

### 3.8. Statistical Analysis

A completely randomized design (CRD) was applied, with heme iron concentration as the level. The response variables included physicochemical, textural, and color characteristics. The results were expressed as mean values ± standard deviation (SD). In the case of finding significance, Tukey’s mean comparison was made with 95% confidence. For the sensory tests and the pivot profile test, the attributes generated semantically were grouped into different categories, a contingency table was generated, and a chi-squared test cell by cell with correspondence analysis was applied. The JAR data and cumulative frequency graphs were made into three categories. The data were processed using the R and XLSTAT 2022 software.

## 4. Conclusions

The enrichment of foods with heme iron concentrate from bovine blood influences the physicochemical, textural and chromatic characteristics of the enriched petipan. The higher the percentage of the heme iron concentrate, the greater the influence on the physicochemical and chromatic characteristics, generating an increase in the protein content and a decrease in color in the upper and lower crust and in the crumb. The percentage of heme concentrate affects the instrumental texture profile, including specifically hardness, fracturability, cohesiveness, elasticity and chewiness, although adhesiveness did not show this behavior. The sensory analysis allowed us to describe the attributes of petipan, characterized as soft, sweet, spongy, strong taste and consistent. Regarding overall liking, the samples without and with one percent heme iron concentrate were described as liking it very much.

## Figures and Tables

**Table 1:** Proximal composition of enriched petipan.

Sample	Ash (g)	Fat (g)	Moisture (g)	Protein (g)	Carbohydrates (g)	Energy (kcal/100 g)
T0	2.145 ± 0.007 ^a,b^	7.525 ± 0.078 ^a^	24.885 ± 0.092 ^a^	9.285 ± 0.050 ^d^	56.160 ± 0.057 ^a^	329.505 ± 0.728 ^a^
T1	2.210 ± 0.014 ^a^	7.365 ± 0.233 ^a^	24.595 ± 0.035 ^b^	9.880 ± 0.113 ^c^	55.950 ± 0.170 ^a^	329.605 ± 0.969 ^a^
T2	2.130 ± 0.014 ^b^	7.270 ± 0.170 ^a^	24.440 ± 0.042 ^b^	10.275 ± 0.035 ^b^	55.885 ± 0.177 ^a^	330.070 ± 0.962 ^a^
T3	2.125 ± 0.035 ^b^	7.435 ± 0.050 ^a^	24.945 ± 0.064 ^a^	10.400 ± 0.028 ^b^	55.095 ± 0.078 ^b^	328.895 ± 0.643 ^a^
T4	2.180 ± 0.014 ^a,b^	7.225 ± 0.134 ^a^	25.030 ± 0.014 ^a^	11.080 ± 0.028 ^a^	54.485 ± 0.078 ^c^	327.285 ± 0.785 ^a^

Values with different letters in the same column show significant differences (*p* ≤ 0.05) according to Tukey’s test.

**Table 2:** Data on the iron content and physical parameters of petipan.

Sample	Iron (mg/kg)	pH	% Acidity (Expressed in Sulfuric Acid)	Bulk Density (g/mL)	Specific Volumen (mL/g)	Porosity (%)
T0	46.600 ± 0.424 ^e^	5.720 ± 0.014 ^a^	0.031 ± 0.002 ^c^	0.436 ± 0.055 ^a^	2.323 ± 0.303 ^a^	8.049 ± 1.362 ^b,c^
T1	61.050 ± 1.202 ^d^	5.615 ± 0.007 ^b^	0.033 ± 0.001 ^c^	0.425 ± 0.032 ^a^	2.362 ± 0.178 ^a^	26.810± 5.190 ^a^
T2	67.440 ± 3.020 ^c^	5.590 ± 0.014 ^b^	0.040 ± 0.001 ^b^	0.419 ± 0.006 ^a^	2.390 ± 0.034 ^a^	16.170 ± 4.870 ^b^
T3	82.350 ± 0.354 ^b^	5.465 ± 0.007 ^c^	0.042 ± 0.001 ^b^	0.416 ± 0.045 ^a^	2.427 ± 0.258 ^a^	13.180 ± 2.690 ^b,c^
T4	93.750 ± 1.202 ^a^	5.465 ± 0.007 ^c^	0.056 ± 0.004 ^a^	0.407 ± 0.051 ^a^	2.493 ± 0.319 ^a^	6.290 ± 2.230 ^c^

Values with different letters in the same column show significant differences (*p* ≤ 0.05) according to Tukey’s test.

**Table 3:** Data on the color of the crust, crumb and base of the petipan.

Sample	L*	a*	b*	C*	h*
	Crust				
T0	53.870 ± 3.010 ^a^	18.195 ± 0.387 ^a^	29.010 ± 2.130 ^a^	57.870 ± 2.580 ^a^	34.422 ± 1.864 ^a^
T1	45.310 ± 2.910 ^b^	14.533 ± 0.282 ^b^	23.270 ± 3.880 ^b^	55.540 ± 2.250 ^ab^	25.770 ± 1.976 ^b^
T2	44.810 ± 2.560 ^b^	12.653 ± 0.497 ^c^	19.120 ± 1.705 ^bc^	56.190 ± 2.080 ^ab^	22.815 ± 1.526 ^b^
T3	45.275 ± 1.591 ^b^	10.563 ± 0.177 ^d^	16.192 ± 0.203 ^cd^	56.810 ± 0.418 ^ab^	19.333 ± 0.236 ^c^
T4	40.477 ± 1.483 ^b^	9.883 ± 0.297 ^d^	13.405 ± 0.724 ^d^	53.572 ± 1.546 ^b^	16.657 ± 0.634 ^c^
	Crumb				
T0	61.270 ± 2.470 ^a^	9.197± 0.426 ^a^	23.523 ± 0.508 ^a^	68.698 ± 0.441 ^a^	25.250 ± 0.616 ^a^
T1	50.085 ± 1.214 ^b^	10.512 ± 0.368 ^b^	19.505 ± 0.364 ^b^	61.683 ± 0.455 ^b^	22.165 ± 0.481 ^b^
T2	44.250 ± 2.080 ^c^	9.408± 0.300 ^b^	16.692 ± 0.869 ^c^	60.962 ± 0.416 ^b^	19.380 ± 0.562 ^c^
T3	43.745 ± 1.012 ^cd^	9.550 ± 0.061 ^b^	15.963 ± 0.138 ^c^	59.102 ± 0.338 ^c^	18.598 ± 0.102 ^cd^
T4	40.493 ± 0.949 ^d^	9.675 ± 0.260 ^b^	14.720± 0.557 ^d^	56.670 ± 0.704 ^d^	17.618 ± 0.574 ^d^
	Base				
T0	40.877 ± 0.662 ^a^	19.403 ± 0.777 ^ab^	26.780 ± 2.760 ^a^	53.037 ± 0.065 ^a^	32.270 ± 1.293 ^a^
T1	36.427 ± 1.111 ^b^	22.807 ± 0.933 ^a^	16.667 ± 0.355 ^b^	36.167 ± 0.696 ^b^	27.910 ± 1.333 ^ab^
T2	33.777 ± 1.269 ^bc^	18.970 ± 2.750 ^ab^	12.270 ± 1.970 ^b^	32.850 ± 1.429 ^b^	22.600 ± 3.330 ^bc^
T3	34.457 ± 1.284 ^b^	20.270 ± 2.660 ^ab^	15.280 ± 1.552 ^b^	28.840 ± 12.310 ^b^	23.850 ± 5.010 ^bc^
T4	31.430 ± 0.212 ^c^	16.903 ± 0.555 ^b^	3.003 ± 1.125 ^c^	10.100 ± 3.940 ^c^	17.193 ± 0.378 ^c^

Values with different letters in the same column show significant differences (*p* ≤ 0.05) according to Tukey’s test.

**Table 4:** Instrumental texture profile (TPA) of petipan.

Sample	Hardness (N)	Adhesiveness (N)	Resilience	Fractureability (N)	Cohesiveness	Elasticity	Chewiness (N)
T0	19.520 ± 2.620 ^b^	0.012 ± 0.003 ^a^	0.140 ± 0.000 ^a^	19.520 ± 2.620 ^b^	0.380 ± 0.014 ^a^	0.785 ± 0.007 ª	5.864 ± 0.918 ^b^
T1	24.508 ± 0.323 ^b^	0.039 ± 0.014 ^a^	0.125 ± 0.007 ^a^	24.508 ± 0.323 ^b^	0.355 ± 0.007 ^ab^	0.740 ± 0.014 ^b^	6.426 ± 0.148 ^b^
T2	25.592 ± 0.121 ^b^	0.032 ± 0.010 ^a^	0.135 ± 0.007 ^a^	25.592 ± 0.121 ^b^	0.360 ± 0.014 ^ab^	0.745 ± 0.007 ^ab^	6.860 ± 0.205 ^ab^
T3	25.180 ± 1.740 ^b^	0.047 ± 0.052 ^a^	0.125 ± 0.007 ^a^	25.180 ± 1.740 ^b^	0.355 ± 0.007 ^ab^	0.760 ± 0.014 ^ab^	6.793 ± 0.179 ^ab^
T4	38.980 ± 1.910 ^a^	0.017 ± 0.001 ^a^	0.095 ± 0.007 ^b^	38.980 ± 1.910 ^a^	0.320 ± 0.014 ^b^	0.720 ± 0.001 ^b^	8.970 ± 0.972 ^a^

Values with different letters in the same column show significant differences (*p* ≤ 0.05) according to Tukey’s test.

**Table 5:** Contingency table of the pivot profile analysis.

Category	Descriptors	T1	T2	T3	T4
Flavor	Acid	48	44	45	45
	Pleasurable	46	47	47	51
	Sour	46	46	46	47
	Bitter	44	46	38	40
	Sweet	67	56	52	63
	Bread flavor	46	45	48	46
	Strong taste	45	45	45	45
	Salty	46	44	45	49
Visual appearance	Dark	16 (−) *	11	10	0 (+) ***
	Bright	48	51	49	49
Texture	Consistent	46	46	44	45
	Hard	34	22	37 (−) *	19 (+) *
	Spongy	49	48	48	48
	Humidity	46	46	47	47
	Dry	43	46	46	46
	Soft	48	51	50	60
Smell	Bread smell	43	44	51	47
	Intense smell	47	48	48	48

(−) and (+) indicate that the observed value is higher or lower than the expected theoretical value (*** *p* < 0.001 and * *p* < 0.05; chi square cell by cell test).

## Data Availability

The data are contained in the article.
